# A systematic review of the role of vitamin insufficiencies and supplementation in COPD

**DOI:** 10.1186/1465-9921-11-171

**Published:** 2010-12-06

**Authors:** Ioanna G Tsiligianni, Thys van der Molen

**Affiliations:** 1 Department of General Practice, University Medical Center Groningen, University of Groningen, Groningen, The Netherlands

## Abstract

**Background:**

Pulmonary inflammation, oxidants-antioxidants imbalance, as well as innate and adaptive immunity have been proposed as playing a key role in the development of COPD. The role of vitamins, as assessed either by food frequency questionnaires or measured in serum levels, have been reported to improve pulmonary function, reduce exacerbations and improve symptoms. Vitamin supplements have therefore been proposed to be a potentially useful additive to COPD therapy.

**Methods:**

A systematic literature review was performed on the association of vitamins and COPD. The role of vitamin supplements in COPD was then evaluated.

**Conclusions:**

The results of this review showed that various vitamins (vitamin C, D, E, A, beta and alpha carotene) are associated with improvement in features of COPD such as symptoms, exacerbations and pulmonary function. High vitamin intake would probably reduce the annual decline of FEV1. There were no studies that showed benefit from vitamin supplementation in improved symptoms, decreased hospitalization or pulmonary function.

## Introduction

COPD in all stages of severity is a very prevalent disease and a great burden for patients and society [1]. In affluent countries COPD is related to smoking over a long period of time, whereas in many other countries it is also related to indoor and outdoor air pollution [1]. The pathology of chronic obstructive pulmonary disease include pulmonary inflammation, oxidants-antioxidants imbalance, protease-antiprotease imbalance, and both innate and adaptive immunity [2,3]. Smoking cessation has been proven to be effective in stopping further deterioration of pulmonary function, reducing symptoms and improving overall health [4]. Smoking cessation however, seems to have only limited influence on the inflammatory process that is associated with COPD. This inflammatory process is probably initiated by oxidative stress and forms the basis of the pathophysiology of COPD [5-8]. Thus the inflammatory process that is associated with COPD seems to be triggered by noxious gasses such as smoking and serious indoor or outdoor air pollution. Oxidative stress caused by these noxious gasses at the level of the epithelium of the bronchial tree might have play a key role in this inflammatory process. It is therefore possible that anti oxidant therapy or an intensive anti oxidant diet could have an influence on the inflammatory process and the progression of COPD. Over the last two decades a number of studies have suggested that COPD risk is associated with vitamins that all have antioxidant properties and with an anti oxidant diet. Low diet-intake of vitamins has been reported to reduce natural defenses and increase the possibility of airway inflammation [9]. Furthermore, a higher intake of fruits and vegetables was associated with a lower risk of COPD, lower mortality and an improvement of spirometric values [10-17].

When levels of vitamins were measured in the serum they were found to be significantly lower in COPD patients than in control subjects [18]. The association of vitamins with pulmonary diseases is further supported by a meta-analysis of 40 studies in patients with asthma. This meta-analysis revealed that relatively low dietary intake of vitamins A and C were associated with statistically significant increased odds of asthma and wheezing [19].

A large number of studies and reviews highlight an association of vitamins with lung function in healthy subjects and COPD patients [20-27]. Recently a randomized controlled trial suggested that a dietary shift to higher antioxidant food intake was associated with improvement in lung function [25]. Furthermore, several studies associate vitamins with a reduction in symptoms, respiratory infections and exacerbations [28-37].

Although for vitamin D the role in respiratory diseases has been clarified through its implication in immunity, for most other vitamins the mechanism of action is less clear [38-42]. Indeed we know that 1,25-dihydroxyvitamin D stimulates both innate and adaptive immunity, in addition to mineralization and calcium homeostasis. Further support on the important role of Vitamin D is given by the fact that it regulates genes that are implicated in apoptosis and cellular proliferation [39], known to be an important step in COPD pathogenesis [42]. Vitamin D has both immunomodulatory and antiiflammatory properties [43]. For vitamin A, B, C, and E, studies highlight their role in COPD risk as well as their connection with COPD outcomes such as symptoms and improvement in spirometric values, without a clear mechanism of action. This article aims to update the knowledge we have about the association between vitamin intake and COPD in outcome measures, as well as to assess the potential role of vitamin supplements.

## Methods

A systematic literature search was performed from 1989 until June 2010 in Pubmed, Embase and Cochrane Collaboration containing the following keywords: COPD in conjunction with smoking, gene polymorphisms, vitamins, FEV1, vitamin C, vitamin E, vitamin D, vitamin A, b-carotene, and vitamin supplements. Further articles were identified from the reference lists of the included articles. In order to be as accurate as possible we included in the present review only studies that have measured serum vitamin levels or used validated food frequency questionnaires to assess the role of vitamins.

Other dietary factors -cured meats, fish, whole grains and alcohol- that have been reported to be associated with the risk of chronic obstructive pulmonary disease or with an increase in symptoms were not included in this review [44-51]. Further we did not examine the influence of caloric intake on COPD. Weight loss and muscle wasting, considered complications of COPD strongly associated with diet, are also not included in the review.

## Results

### Methods of assessing vitamin status: which is best?

The literature reports two essentially different ways to measure vitamins: Serum levels and Food Frequency Questionnaires (FFQ). Both measures have their advantages and disadvantages.

#### Serum levels of vitamins

The assessment of serum levels of vitamins can have the advantage of being more objective than patient's reported intake. However, serum level assessment of vitamins has the disadvantage that they represent the more recent intake, and for some vitamins such as vitamin C, the levels in peripheral blood are not representative of intake and do not change accordingly [19,52].

#### Food Frequency questionnaire

Food frequency questionnaires on the other hand present a large heterogeneity with differences in assessing periods (from 1 day to two years), number of items on food questionnaires (ranging from 44 to 350) and use of portion size questions [53]. Further, it has been suggested that FFQs do not always detect weak associations [54]. Regarding vitamin C intake large differences were found between FFQs that used portion size questions instead of using standard portions [53]. Another problem regarding FFQ use is that it is difficult to determine which particular vitamin is associated with COPD and if it is the vitamins in fruits and vegetables that are associated with COPD, or another confounding nutrient. Resveratrol for example, a phenolic antioxidant is present in many fruits and is associated with anti-inflammatory activity [55,56]. Therefore it is not clear if in some cases, the vitamins have the beneficial effect, or other nutrients such as resveratrol with antioxidant and anti-inflammatory properties.

Another important obstacle in FFQ is that an assessment of vitamin D from food intake would lead to an incorrect estimation in for example Mediterranean countries where there is a high skin synthesis because of the sunlight.

We found 14 references [31,57-69] that assessed the relationship between a vitamin rich diet as assessed by a FFQ and the subsequent improvement of spirometric values and symptoms. Twelve studies [32,33,35,52,63,70-76] measured serum levels of vitamins. For the above mentioned reasons, and in order to have a more precise overview, we decided to include both FFQ and serum level studies.

### Food intake patterns: Effect of vitamins on the risk of developing COPD and associated mortality

Varraso et al in a study of 72,043 women identified 754 cases of newly diagnosed COPD [44]. In this study a healthy diet (fruit, vegetables, fish, whole-grain products) was compared with a Western diet (refined grains, cured and red meats, desserts, French fries). The healthy diet was associated with a lower risk of COPD [44]. This could be considered to be due to the overall diet, or indicate a possible positive effect of vitamins on COPD risk, as fruits are considered sources rich with vitamins. From the same author another study comparing the same patterns of diet showed the same results in 111 self-reported cases of newly diagnosed COPD in men [46]. Celik et al used a food frequency questionnaire and found that the consumption of fruits and vegetables was significantly lower in COPD patients compared to the control group [77]. Fruit intake was related to a lower 25-year incidence of chronic bronchitis and emphysema [57] as well as spirometry improvement [78]. A recent randomized controlled trial has shown that a dietary shift to more anti-oxidant foods such as fruits and vegetables is associated with improvement in lung function [25].

These studies showed a protective role of vitamins against COPD but did not measure vitamins in serum or with a FFQ. Therefore conclusions about the special role of any vitamin in COPD could not be obtained.

### Vitamin D and COPD

Vitamin D is extremely important for the human body. It has a significant role in bone mineralization, in calcium and phosphorous absorption, and is important in the immune system [39,40]. It's most important role however is in bone structure development and bone turn over, as a low vitamin D level is directly associated with osteoporosis. The main sources of vitamin D are skin synthesis and diet. The precursor form is 7-dehydrocholesterol which with UVB is transformed to vitamin D3. Vitamin D3 is transported via the D-binding protein (DBP) to the liver where with hydroxylation reactions transforms to 25(OH)D3 and which is transported again by DBP to the kidneys where it takes its active form of 1,25(OH)2D3. 25OHD3 can also be transformed in 1,25(OH)2D3 in the immune cells [42]. Some studies showed a DBP Gc-1F allele presence that was higher in COPD patients [79,80] and Schellenberg et al found that the Gc2 homozygous genotype was protective for COPD [81]. Other polymorphisms associated with vitamin D binding protein gene are related to clinical differences in families with alpha-1-antitrypsin deficiency [82].

COPD is characterized by inflammation induced by macrophages and neutrophils (innate immunity). COPD is considered a disease where proinflammattory cytokines are increased and has a Th2 response with a predominance of CD8 lymphocytes (adaptive immunity). 1,25-dihydroxyvitamin D stimulates innate immunity probably due to activation of cathelicidin (antimicrobial peptides) to enhance the bacterial killing via Toll-like receptors [83,84]. Vitamin D receptors (VDR) are present in various cells of both innate (ie.macrophages) and adaptive immunity (i.e.T and B cells). Vitamin D is able to modulate both types of immunity therefore minimizing inflammation [85]. Vitamin D in general is involved in modulating cellular proliferation, suppressing TH cells, [86], downregulating cytokines such as IL-2 [87], as well as in the inhibition of dendritic cells [88], all of which are known to be important in the COPD pathway. Regarding respiratory function, vitamin D plays a significant role in airway remodeling through the inhibition of TNFa and enhancement of Il-10 in immune cells [39]. Vitamin D also seems to play a role as an alternative treatment strategy to reverse glucocorticoid resistance through its ability to restore IL-10 response [89]. This is important since glucorticoid resistance is a pivotal barrier to the anti inflammatory treatment of COPD.

Patients with COPD have an increased prevalence of osteoporosis (from 9-69%) and osteopenia (from 27-67%) [90-93]. Malnutririon and low vitamin D levels could be a cause of this higher prevalence [91,94]. The majority of COPD patients have vitamin D deficiency [39,41,95-97] therefore vitamin D supplementation in patients with COPD has been proposed [40].

Black et al reported that higher vitamin D levels were associated with better lung function [72]. In this study that used cross-sectional data from the Third National Health and Nutrition Examination Survey 14.091 people aged >20 years were included. The mean difference between the highest and the lowest quintile of 25-hydroxyvitamin D serum concentration was 126 ml in FEV 1, and 172 ml for FVC after adjustment for factors that affect lung function (age, gender, smoking, etc) [72] (Table 1).

**Table 1:** Studies connecting spirometric values or incidence of respiratory infections with Vitamin D.

Vitamin D-Ref	No of participants	FFQ or plasma levels	Results
[33]	18.883	Plasma levels	Lower 25(OH)D levels were independently associated with recent URTI (odds ratio [OR], 1.36-1,24). The association between 25(OH)D level and URTI was stronger in patients with chronic obstructive pulmonary disease odds ratio; 2.26.

[35]	800	Plasma levels	Subjects with serum 25(OH)D concentrations < 40 nmol/L (n = 24) had significantly (P = 0.004) more days of absence from duty due to respiratory infection (median: 4; quartile 1-quartile 3: 2-6) than did control subjects (2; 0-4; n = 628; incidence rate ratio 1.63; 95% CI: 1.15, 2.24).

[72]	14.091	Plasma levels	The mean difference between the highest and the lowest quintile of 25-hydrocyvitamin D serum concentration was 126 mL (SE:22 mL) in FEV 1, and 172 mL (SE:22 mL) for FVC.

Vitamin D insufficiency has been reported to be associated with an increased incidence of chronic respiratory infections [29,33-35]. There are some studies that also suggest that low serum 25-hydroxy vitamin D levels are associated with upper and lower respiratory tract infection [33-35]. In one large cross sectional study with 18.883 participants, this association was stronger in COPD patients [33] (Table 1). Ebstein Barr virus infection, which is often found in COPD patients, is also associated with low levels of vitamin D [98,99]. Liou et al reported a relation between Toll-like receptors, external triggers and vitamin D-mediated innate immunity, and suggested that differences in the ability of human populations to produce vitamin D may contribute to susceptibility to microbial infections [100].

Finally, Vitamin D could play an important role as an antioxidant therapy, not only for the significant improvement in spirometric values, but also because it has been proposed as a novel treatment to cachexia and sarcopenia in COPD patients [101].

### Vitamin C and E

The role of vitamin C (also known as ascorbate or L-ascorbic acid) in the human body is essential. It has antioxidant properties, is involved in various metabolic reactions, and some studies report it also plays a role in the immune system [102,103]. It is considered important for the maintenance of the connective tissue and bone remodeling [102]. Vitamin E has antioxidant properties as well, and has been reported to have a protective role in the prevention of atherosclerosis and carcinogenesis [104].

In one study that included 3 European Countries a trend (P < 0.05) of lower COPD mortality was observed with vitamin E intake, while no trend was found with vitamin C after adjustment for age, smoking and country [16]. Higher levels of vitamin C and E in both serum and FFQ in healthy subjects were associated with an increase in FEV1 and FVC [31,32,58-66,69,70]. More details are depicted in Table 2. In one study an increase of 20 micromol/Lt in plasma vitamin C concentration was associated with a 13% reduction in the risk of developing obstructive airway disease OR: 0.87 (CI:0.77-0.98) [75].

**Table 2:** Studies connecting Vitamin C, E, A, alpha and beta-carotene with spirometric values improvement.

Vitamin	FFQ studies	Plasma levels studies	Improvement in spirometric values	No association with spirometric values
Vit C	^31,58,59,60,61, 62,63,65,66^	^32,52, 63,69,70^	**Serum**:FEV1 improvement in ml from 17-94 ml and FVC improvement from 16.4-94 ml for an SD variation **FFQ**: FEV1 improvement in ml from 37-53 ml and FVC improvement from 23.3-79 ml for an SD variation	^52^

Vit E	^31,58,59,61, 62,64,65^	^32,69,70^	**Serum**: An SD increase in plasma levels of vitamin E had a median range of FEV1 increase in ml from 12-59.3 ml **FFQ**: An SD increase had a median range of FEV1 increase in ml from 20.1-93 ml and for FVC from 23.1 -54 ml, respectivelly	^31,58,61^

Vit A	^61,68^	^32,70^	^32,70^ **Serum**:Improvement in FEV1 ranges from 22-31.2 ml	^61,68^

b-carotene	^31,57, 63,65,66,69 ^	^32,69, 70, 76 ^	**Serum: **Improvement in FEV1 ranges from 11-107 ml, FVC 147 ml **FFQ: **Improvement in FEV1 = 60 ml, FVC= 75 ml	^57^

a-carotene		^70,71^	^70,71^ **Serum**: Improvement in FEV1 for one SD increase 23.7 ml^70^. Subjects in the fifth quintile of serum beta-carotene had a 195 ml (95% confidence interval [95% CI]: 40 to 351 ml) higher and those in the fifth quintile of alpha-carotene had a 257 ml (95% CI: 99 to 414 ml) higher FEV(1) compared with subjects in the first quintile of these carotenoids^71^.	

Studies regarding the role of vitamin C and E in respiratory symptoms showed that low levels were associated with more wheezing, phlegm production and dyspnea [28,31,32,36,37]. Tug et al found both vitamin E and vitamin A levels were significantly lower during exacerbations of COPD than in patients with stable COPD [30].

Takkouche et al, in 1667 cases of the common cold in the general population suggested that intake of vitamin C and zinc was not related to the occurrence of common cold [105]. Nevertheless vitamin C decreases the duration of common cold symptoms which might be important in patients with COPD [106].

### Vitamin A and B

Vitamin A (retinol and carotens) plays an important role in several functions of the human body including vision, bone and skin health, and further has an innate antioxidant activity. Vitamin B is involved in various steps of metabolism and enhances immunity. High levels of vitamin A, b-carotene and/or alpha-carotene were associated with increase in FEV1 and FVC in most of the studies [31,32,63,65,66,69-71,76] although there are some exceptions [57,61,68]. More details are presented in Table 2. High serum beta carotene levels in a general population sample of 523 subjects were associated with the expression of a gene polymorphism that connected with a slower FEV1 decline [107].

Hirayama et al reported that the highest level of intake of vitamin A resulted in a 52% (p = 0.008) reduction in COPD risk [108] while in another study the risk for COPD was associated with lower levels of plasma vitamin A (p < 0.01)[108].

Fimognari et al reported lower levels of folate and vitamin B12 in COPD patients, resulted in an increased plasma level of total homocysteine, a known cardiovascular risk factor [109].

Regarding the role of b-carotene in respiratory symptoms, two studies showed a beneficial association with cough [32,36] and one study showed no correlation with symptoms, except for wheezing [31].

### Vitamin supplementation

Antioxidant supplementation has been proposed to be helpful in patients with COPD as a way to reduce oxidative stress and inflammation, and improve spirometric values [24]. Multivitamin supplementation has been reported to be popular for patients with COPD especially among older patients [110].

Seven studies reported the effect of vitamin supplements on several outcomes of COPD [18,36,111-115]. All these studies showed a large heterogeneity regarding: which vitamins had been supplemented, the dosage and the duration of the vitamin supplementation, ranging from 4 weeks to 5 years [111-115]. Secondly, different outcomes were measured such as spirometric values, symptoms and exercise capacity. A randomized controlled trial in high-risk individuals for cardiovascular events that received antioxidant vitamins (vitamin C, E and b-carotene) supplementation for 5 years failed to identify any improvement in 5-year mortality and in spirometric values or hospitalization due to COPD. However this study excluded patients with severe COPD [115]. More details are depicted in Table 3. Also a cohort study of 77,719 participants using multi-vitamin supplements was not related to the total mortality [116].

**Table 3:** Vitamin supplementation and COPD outcome measured.

Reference	Suplementation	No of patients	Effect
[18]	Supplementation E and C. 10 of 21 COPD patients were given vitamin E (200 UI/day) and vitamin C (500 mg/day) for 1 month.	21 COPD and 10 controls.	The exercise time increased significantly in the 10 COPD patients who were treated (exercise time 6.4+1.8 vs 8.7+2.1 min, p = 0.01). (Bruce protocol-graded treadmill exercise test).

[36]	Supplementation alpha-tocopherol (50 mg/d) and beta-carotene (20 mg/d) supplementation, for 5-8 years.	29.133 people (Cancer prevention study)	The supplementation did not affected the reccurence or incidence of chronic cough, phlegm or dyspnea. Relative risk for the above mentioned symptoms arround 1 with or without supplementation.

[111]	Vit E supplementation. 400 IU daily for 12 weeks.	30 COPD patients	Spirometric measurements. Changed not significant either on day 1 or after 12 weeks of vitamin E supplementation.

[112]	Vit E supplementation Patients were divided into two groups: group A- placebo group (n = 14), receiving only standard therapy, and group B- vitamin E-supplemented group (n = 10), receiving 400 IU of vitamin E capsules twice daily in addition to standard therapy, for 8 weeks.	24 COPD patients.	There was a similar degree of lung function and clinical improvement in both groups.

[113]	Vit C and E. Patients were randomly assigned to placebo (n = 8), 400 mg/day vitamin E (E400, n = 9), 200 mg/day vitamin E (E200, n = 9), or 250 mg/day vitamin C (C250, n = 9) for 12 weeks.	35 COPD patients	No improvement in lung function after 12 weeks of supplementation.

[114]	Vit A supplementation for 30 days. (healthy nonsmokers (n = 7), healthy smokers (n = 7), mild chronic obstructive pulmonary disease (COPD-mild) patients (n = 9), COPD-moderate-severe patients (n = 7), and COPD-moderate-severe patients with exacerbation (+ex;n = 6)	36 people-21 COPD n = 6).	Improvement in lung function mean increase for 1-s forced expiratory volume (FEV1) = 22.9% in the COPD-vitamin A group.

[115]	Supplementation 600 mg vitamin E, 250 mg vitamin C, and 20 mg β-carotene daily 5-year treatment period. All participants randomly allocated to receive vitamin supplementation or placebo.	20 536 UK adults (aged 40-80) with coronary disease, other occlusive arterial disease, or diabetes	No significant differences were observed between the treatment groups in forced expiratory volume during one second (FEV_1_: 2·06 L vitamin-allocated *vs *2·06 L placebo-allocated; difference 0·00 L [SE 0·01]) or in forced vital capacity (FVC: 2·83 L *vs *2·82 L; difference 0·01 L [SE 0·01]). Nor were significant differences observed in the numbers of participants hospitalised for chronic obstructive pulmonary disease or asthma (149 [1·5%] *vs *133 [1·3%]) or for any other non-neoplastic respiratory cause (641 [6·2%] *vs *642 [6·3%]). .

Little is known regarding the prevention of upper respiratory tract infections after supplementation of Vitamin D although some studies report a trend for improvement [117] but some others do not confirm that (ranges of OR = 0.77-0.95) [118,119].

## Conclusion

The results of this review show that intake of various vitamins are associated with improvement in features of COPD such as symptoms, exacerbations and pulmonary function. Increased vitamin intake could probably reduce the annual decline of FEV1. Although the mechanisms behind these effects are often not clear, this might open possibilities to develop drugs that modify or prevent COPD. Dietary interventions directed towards high vitamin intake might be an additional approach towards COPD management. Although there are many studies that associate vitamins with improvement in lung function tests, there is no clear evidence of the benefit of vitamin supplements. Most studies regarding supplements showed no benefit of multivitamin supplemention in symptoms, spirometric function or hospitalization for COPD.

This review suggests that future work is needed with prospective randomized controlled trials, that would explore the role of vitamins as well as the effectiveness of vitamin supplements on outcomes such as symptoms spirometric values, health status, risk of development of COPD and exacerbations rates.

## Competing interests

The authors declare that they have no competing interests.

## Authors' contributions

Both authors (IGT, TvdM) wrote and revised the manuscript, and approved the final version.

## References

[B1] Global Initiative for Chronic Obstructive Lung Disease http://www.goldcopd.com (assessed June 2010)

[B2] Saetta M Turato G Maestrelli P Mapp CE Fabbri LM Cellular and structural bases of chronic obstructive pulmonary disease Am J Respir Crit Care Med 2001 163 1304 1309 11371392 10.1164/ajrccm.163.6.2009116

[B3] Grashoff WF Sont JK Sterk PJ Hiemstra PS de Boer WI Stolk J Han J van Krieken JM Chronic obstructive pulmonary disease: role of bronchiolar mast cells and macrophages Am J Pathol 1997 151 1785 1790 9403729 PMC1858350

[B4] Willemse BW Postma DS Timens W ten Hacken NH The impact of smoking cessation on respiratory symptoms, lung function, airway hyperresponsiveness and inflammation Eur Respir J 2004 23 3 464 76 10.1183/09031936.04.00012704 15065840

[B5] Rahman I Adcock IM Oxidative stress and redox regulation of lung inflammation in COPD Eur Respir J 2006 28 1 219 42 10.1183/09031936.06.00053805 16816350

[B6] Siafakas NM Tzortzaki EG Few smokers develop COPD. Why? Respir Med 2002 96 8 615 24 10.1053/rmed.2002.1318 12195843

[B7] Brigham KL Role of free radicals in lung injury Chest 1986 89 6 859 63 10.1378/chest.89.6.859 3519109

[B8] Sanguinetti CM Oxidant/antioxidant imbalance: role in the pathogenesis of COPD Respiration 1992 59 Suppl 1:20 3 10.1159/000196098 1579727

[B9] Heffner JE Repine JE Pulmonary strategies of antioxidant defense Am Rev Respir Dis 1989 140 2 531 54 2669581 10.1164/ajrccm/140.2.531

[B10] Brug J Schols A Mesters I Dietary change, nutrition education and chronic obstructive pulmonary disease Patient Educ Couns 2004 52 3 249 57 10.1016/S0738-3991(03)00099-5 14998594

[B11] Watson L Margetts B Howarth P Dorward M Thompson R Little P The association between diet and chronic obstructive pulmonary disease in subjects selected from general practice Eur Respir J 2002 20 2 313 8 10.1183/09031936.02.00256402 12212961

[B12] Tabak C Feskens EJ Heederik D Kromhout D Menotti A Blackburn HW Fruit and fish consumption: a possible explanation for population differences in COPD mortality (The Seven Countries Study) Eur J Clin Nutr 1998 52 11 819 25 10.1038/sj.ejcn.1600653 9846595

[B13] Tabak C Arts IC Smit HA Heederik D Kromhout D Chronic obstructive pulmonary disease and intake of catechins, flavonols, and flavones: the MORGEN Study Am J Respir Crit Care Med 2001 164 1 61 4 11435239 10.1164/ajrccm.164.1.2010025

[B14] Denny SI Thompson RL Margetts BM Dietary factors in the pathogenesis of asthma and chronic obstructive pulmonary disease Curr Allergy Asthma Rep 2003 3 2 130 6 10.1007/s11882-003-0025-6 12562552

[B15] Carey IM Strachan DP Cook DG Effects of changes in fresh fruit consumption on ventilatory function in healthy British adults Am J Respir Crit Care Med 1998 158 3 728 33 9730997 10.1164/ajrccm.158.3.9712065

[B16] Walda IC Tabak C Smit HA Räsänen L Fidanza F Menotti A Nissinen A Feskens EJ Kromhout D Diet and 20-year chronic obstructive pulmonary disease mortality in middle-aged men from three European countries Eur J Clin Nutr 2002 56 7 638 43 10.1038/sj.ejcn.1601370 12080403

[B17] Strachan DP Cox BD Erzinclioglu SW Walters DE Whichelow MJ Ventilatory function and winter fresh fruit consumption in a random sample of British adults Thorax 1991 46 9 624 9 10.1136/thx.46.9.624 1948789 PMC463343

[B18] Agacdiken A Basyigit I Ozden M Yildiz F Ural D Maral H Boyaci H Ilgazli A Komsuoglu B The effects of antioxidants on exercise-induced lipid peroxidation in patients with COPD Respirology 2004 9 1 38 42 10.1111/j.1440-1843.2003.00526.x 14982600

[B19] Allen S Britton JR Leonardi-Bee JA Association between antioxidant vitamins and asthma outcome measures: systematic review and meta-analysis Thorax 2009 64 7 610 9 10.1136/thx.2008.101469 19406861

[B20] Schünemann HJ Freudenheim JL Grant BJ Epidemiologic evidence linking antioxidant vitamins to pulmonary function and airway obstruction Epidemiol Rev 2001 23 2 248 67 12192736 10.1093/oxfordjournals.epirev.a000805

[B21] Sridhar MK Nutrition and lung health Proc Nutr Soc 1999 58 2 303 8 10466171 10.1017/s0029665199000415

[B22] Smit HA Chronic obstructive pulmonary disease, asthma and protective effects of food intake: from hypothesis to evidence? Respir Res 2001 2 5 261 4 10.1186/rr65 11686892 PMC59508

[B23] Smit HA Grievink L Tabak C Dietary influences on chronic obstructive lung disease and asthma: a review of the epidemiological evidence Proc Nutr Soc 1999 58 2 309 19 10466172 10.1017/s0029665199000427

[B24] Romieu I Trenga C Diet and obstructive lung diseases Epidemiol Rev 2001 23 2 268 87 12192737 10.1093/oxfordjournals.epirev.a000806

[B25] Keranis E Makris D Rodopoulou P Martinou H Papamakarios G Daniil Z Zintzaras E Gourgoulianis KI Impact of dietary shift to higher antioxidant foods in COPD: A Randomized Trial Eur Respir J 2010 in press 20150206 10.1183/09031936.00113809

[B26] Schünemann HJ Grant BJ Freudenheim JL Muti P Browne RW Drake JA Klocke RA Trevisan M The relation of serum levels of antioxidant vitamins C and E, retinol and carotenoids with pulmonary function in the general population Am J Respir Crit Care Med 2001 163 5 1246 55 11316666 10.1164/ajrccm.163.5.2007135

[B27] Kelly FJ Vitamins and respiratory disease: antioxidant micronutrients in pulmonary health and disease Proc Nutr Soc 2005 64 4 510 26 10.1079/PNS2005457 16313695

[B28] Schwartz J Weiss ST Dietary factors and their relation to respiratory symptoms. The Second National Health and Nutrition Examination Survey Am J Epidemiol 1990 132 1 67 76 2356815 10.1093/oxfordjournals.aje.a115644

[B29] Zasloff M Fighting infections with vitamin D Nat Med 2006 12 4 388 90 10.1038/nm0406-388 16598282

[B30] Tug T Karatas F Terzi SM Antioxidant vitamins (A, C and E) and malondialdehyde levels in acute exacerbation and stable periods of patients with chronic obstructive pulmonary disease Clin Invest Med 2004 27 3 123 8 15305803

[B31] Grievink L Smit HA Ocké MC van 't Veer P Kromhout D Dietary intake of antioxidant (pro)-vitamins, respiratory symptoms and pulmonary function: the MORGEN study Thorax 1998 53 3 166 71 10.1136/thx.53.3.166 9659349 PMC1745167

[B32] Kelly Y Sacker A Marmot M Nutrition and respiratory health in adults: findings from the health survey for Scotland Eur Respir J 2003 21 4 664 71 10.1183/09031936.03.00055702 12762354

[B33] Ginde AA Mansbach JM Camargo CA Jr Association between serum 25-hydroxyvitamin D level and upper respiratory tract infection in the Third National Health and Nutrition ExaminationSurvey Arch Intern Med 2009 169 4 384 90 10.1001/archinternmed.2008.560 19237723 PMC3447082

[B34] Karatekin G Kaya A Salihoğlu O Balci H Nuhoğlu A Association of subclinical vitamin D deficiency in newborns with acute lower respiratory infection and their mothers Eur J Clin Nutr 2009 63 4 473 7 10.1038/sj.ejcn.1602960 18030309

[B35] Laaksi I Ruohola JP Tuohimaa P Auvinen A Haataja R Pihlajamäki H Ylikomi T An association of serum vitamin D concentrations < 40 nmol/L with acute respiratory tract infection in young Finnish men Am J Clin Nutr 2007 86 3 714 7 17823437 10.1093/ajcn/86.3.714

[B36] Rautalahti M Virtamo J Haukka J Heinonen OP Sundvall J Albanes D Huttunen JK The effect of alpha-tocopherol and beta-carotene supplementation on COPD symptoms Am J Respir Crit Care Med 1997 156 5 1447 52 9372659 10.1164/ajrccm.156.5.96-11048

[B37] Bodner C Godden D Brown K Little J Ross S Seaton A Antioxidant intake and adult-onset wheeze: a case-control study. Aberdeen WHEASE Study Group Eur Respir J 1999 13 1 22 30 10.1183/09031936.99.13102299 10836318

[B38] Chishimba L Thickett DR Stockley RA Wood AM The vitamin D axis in the lung: a key role for vitamin D-binding protein Thorax 2010 65 5 456 62 10.1136/thx.2009.128793 20435872

[B39] Hughes DA Norton R Vitamin D and respiratory health Clin Exp Immunol 2009 158 1 20 5 10.1111/j.1365-2249.2009.04001.x 19737226 PMC2759054

[B40] Janssens W Lehouck A Carremans C Bouillon R Mathieu C Decramer M Vitamin D beyond bones in chronic obstructive pulmonary disease: time to act Am J Respir Crit Care Med 2009 179 8 630 6 10.1164/rccm.200810-1576PP 19164701

[B41] Wright RJ Make no bones about it: increasing epidemiologic evidence links vitamin D to pulmonary function and COPD Chest 2005 128 6 3781 3 10.1378/chest.128.6.3781 16354841

[B42] Holick MF Vitamin D deficiency N Engl J Med 2007 357 3 266 81 10.1056/NEJMra070553 17634462

[B43] Adams JS Hewison M Unexpected actions of vitamin D: new perspectives on the regulation of innate and adaptive immunity Nat Clin Pract Endocrinol Metab 2008 4 2 80 90 10.1038/ncpendmet0716 18212810 PMC2678245

[B44] Varraso R Fung TT Barr RG Hu FB Willett W Camargo CA Jr Prospective study of dietary patterns and chronic obstructive pulmonary disease among US women Am J Clin Nutr 2007 86 2 488 95 17684223 10.1093/ajcn/86.2.488 PMC2643338

[B45] Tabak C Smit HA Heederik D Ocké MC Kromhout D Diet and chronic obstructive pulmonary disease: independent beneficial effects of fruits, whole grains, and alcohol (the MORGEN study) Clin Exp Allergy 2001 31 5 747 55 10.1046/j.1365-2222.2001.01064.x 11422134

[B46] Varraso R Fung TT Hu FB Willett W Camargo CA Prospective study of dietary patterns and chronic obstructive pulmonary disease among US men Thorax 2007 62 9 786 91 10.1136/thx.2006.074534 17504819 PMC2117325

[B47] Varraso R Jiang R Barr RG Willett WC Camargo CA Jr Prospective study of cured meats consumption and risk of chronic obstructive pulmonary disease in men Am J Epidemiol 2007 166 12 1438 45 10.1093/aje/kwm235 17785711 PMC2573990

[B48] Varraso R Fung TT Barr RG Hu FB Willett W Camargo CA Jr Prospective study of dietary patterns and chronic obstructive pulmonary disease among US women Am J Clin Nut r 2007 86 2 488 95 10.1093/ajcn/86.2.488 PMC2643338 17684223

[B49] Jiang R Paik DC Hankinson JL Barr RG Cured meat consumption, lung function, and chronic obstructive pulmonary disease among United States adults Am J Respir Crit Care Med 2007 175 8 798 804 10.1164/rccm.200607-969OC 17255565 PMC1899290

[B50] Butler LM Koh WP Lee HP Tseng M Yu MC London SJ Singapore Chinese Health Study. Prospective study of dietary patterns and persistent cough with phlegm among Chinese Singaporeans Am J Respir Crit Care Med 2006 173 3 264 70 10.1164/rccm.200506-901OC 16239624 PMC1447591

[B51] Butler LM Koh WP Lee HP Yu MC London SJ Dietary fiber and reduced cough with phlegm: a cohort study in Singapore Am J Respir Crit Care Med 2004 170 3 279 87 10.1164/rccm.200306-789OC 15117740

[B52] Cook DG Carey IM Whincup PH Papacosta O Chirico S Bruckdorfer KR Walker M Effect of fresh fruit consumption on lung function and wheeze in children Thorax 1997 52 7 628 33 10.1136/thx.52.7.628 9246135 PMC1758609

[B53] Molag ML de Vries JH Ocké MC Dagnelie PC van den Brandt PA Jansen MC van Staveren WA van't Veer P Design characteristics of food frequency questionnaires in relation to their validity Am J Epidemiol 2007 166 12 1468 78 10.1093/aje/kwm236 17881382

[B54] Schatzkin A Kipnis V Carroll RJ Midthune D Subar AF Bingham S Schoeller DA Troiano RP Freedman LS A comparison of a food frequency questionnaire with a 24-hour recall for use in an epidemiological cohort study: results from the biomarker-based Observing Protein and Energy Nutrition (OPEN) study Int J Epidemiol 2003 32 6 1054 62 10.1093/ije/dyg264 14681273

[B55] Olas B Wachowicz B Resveratrol, a phenolic antioxidant with effects on blood platelet functions Platelets 2005 16 5 251 60 10.1080/09537100400020591 16011975

[B56] Sharafkhaneh A Velamuri S Badmaev V Lan C Hanania N The potential role of natural agents in treatment of airway inflammation Ther Adv Respir Dis 2007 1 2 105 20 10.1177/1753465807086096 19124352

[B57] Miedema I Feskens EJ Heederik D Kromhout D Dietary determinants of long-term incidence of chronic nonspecific lung diseases. The Zutphen Study Am J Epidemiol 1993 138 1 37 45 8333425 10.1093/oxfordjournals.aje.a116775

[B58] Morabia A Sorenson A Kumanyika SK Abbey H Cohen BH Chee E Vitamin A, cigarette smoking, and airway obstruction Am Rev Respir Dis 1989 140 5 1312 6 2817593 10.1164/ajrccm/140.5.1312

[B59] Britton JR Pavord ID Richards KA Knox AJ Wisniewski AF Lewis SA Tattersfield AE Weiss ST Dietary antioxidant vitamin intake and lung function in the general population Am J Respir Crit Care Med 1995 151 5 1383 7 7735589 10.1164/ajrccm.151.5.7735589

[B60] Ness AR Khaw KT Bingham S Day NE Vitamin C status and respiratory function Eur J Clin Nutr 1996 50 9 573 9 8880036

[B61] McKeever TM Scrivener S Broadfield E Jones Z Britton J Lewis SA Prospective study of diet and decline in lung function in a general population Am J Respir Crit Care Med 2002 165 9 1299 303 10.1164/rccm.2109030 11991883

[B62] Dow L Tracey M Villar A Coggon D Margetts BM Campbell MJ Holgate ST Does dietary intake of vitamins C and E influence lung function in older people? Am J Respir Crit Care Med 1996 154 5 1401 4 8912755 10.1164/ajrccm.154.5.8912755

[B63] Hu G Zhang X Chen J Peto R Campbell TC Cassano PA Dietary vitamin C intake and lung function in rural China Am J Epidemiol 1998 148 6 594 9 9753014 10.1093/oxfordjournals.aje.a009685

[B64] Butland BK Fehily AM Elwood PC Diet, lung function, and lung function decline in a cohort of 2512 middle aged men Thorax 2000 55 2 102 8 10.1136/thorax.55.2.102 10639525 PMC1745677

[B65] Tabak C Smit HA Räsänen L Fidanza F Menotti A Nissinen A Feskens EJM Heederik D Kromhout D Dietary factors and pulmonary function: a cross-sectional study in middle aged men from three European countries Thorax 1999 54 1021 1026 10.1136/thx.54.11.1021 10525562 PMC1745389

[B66] Ochs-Balcom HM Grant BJ Muti P Sempos CT Freudenheim JL Browne RW McCann SE Trevisan M Cassano PA Iacoviello L Schünemann HJ Antioxidants, oxidative stress, and pulmonary function in individuals diagnosed with asthma or COPD Eur J Clin Nutr 2006 60 8 991 9 10.1038/sj.ejcn.1602410 16482071

[B67] Schwartz J Weiss ST Relationship between dietary vitamin C intake and pulmonary function in the First National Health and Nutrition Examination Survey (NHANES I) Am J Clin Nutr 1994 59 1 110 4 8279390 10.1093/ajcn/59.1.110

[B68] Shahar E Folsom AR Melnick SL Tockman MS Comstock GW Shimakawa T Higgins MW Sorlie PD Szklo M Does dietary vitamin A protect against airway obstruction? The Atherosclerosis Risk in Communities (ARIC) Study Investigators Am J Respir Crit Care Med 1994 150 4 978 82 7921473 10.1164/ajrccm.150.4.7921473

[B69] Hu G Cassano PA Antioxidant nutrients and pulmonary function: the Third National Health and Nutrition Examination Survey (NHANES III) Am J Epidemiol 2000 151 975 981 10853636 10.1093/oxfordjournals.aje.a010141

[B70] McKeever TM Lewis SA Smit HA Burney P Cassano PA Britton J A multivariate analysis of serum nutrient levels and lung function Respir Res 2008 9 67 10.1186/1465-9921-9-67 18823528 PMC2565672

[B71] Grievink L de Waart FG Schouten EG Kok FJ Serum carotenoids, alpha-tocopherol, and lung function among Dutch elderly Am J Respir Crit Care Med 2000 161 3 Pt 1 790 5 10712323 10.1164/ajrccm.161.3.9904040

[B72] Black PN Scragg R Relationship between serum 25-hydroxyvitamin d and pulmonary function in the third national health and nutrition examination survey Chest 2005 128 6 3792 8 10.1378/chest.128.6.3792 16354847

[B73] Pearson P Britton J McKeever T Lewis SA Weiss S Pavord I Fogarty A Lung function and blood levels of copper, selenium, vitamin C and vitamin E in the general population Eur J Clin Nutr 2005 59 9 1043 8 10.1038/sj.ejcn.1602209 16015272

[B74] Førli L Pedersen JI Bjørtuft O Blomhoff R Kofstad J Boe J Vitamins A and E in serum in relation to weight and lung function in patients with advanced pulmonary disease Int J Vitam Nutr Res 2002 72 6 360 8 12596500 10.1024/0300-9831.72.6.360

[B75] Sargeant LA Jaeckel A Wareham NJ Interaction of vitamin C with the relation between smoking and obstructive airways disease in EPIC Norfolk. European Prospective Investigation into Cancer and Nutrition Eur Respir J 2000 16 3 397 403 10.1034/j.1399-3003.2000.016003397.x 11028650

[B76] Grievink L Smit HA Veer P Brunekreef B Kromhout D Plasma concentrations of the antioxidants beta-carotene and alpha-tocopherol in relation to lung function Eur J Clin Nutr 1999 53 10 813 7 10.1038/sj.ejcn.1600854 10556989

[B77] Celik F Topcu F Nutritional risk factors for the development of chronic obstructive pulmonary disease (COPD) in male smokers Clin Nutr 2006 25 6 955 61 10.1016/j.clnu.2006.04.006 16782241

[B78] Strachan DP Cox BD Erzinclioglu SW Walters DE Whichelow MJ Ventilatory function and winter fresh fruit consumption in a random sample of British adults Thorax 1991 46 9 624 9 10.1136/thx.46.9.624 1948789 PMC463343

[B79] Ito I Nagai S Hoshino Y Muro S Hirai T Tsukino M Mishima M Risk and severity of COPD is associated with the group-specific component of serum globulin 1F allele Chest 2004 125 1 63 70 10.1378/chest.125.1.63 14718422

[B80] Ishii T Keicho N Teramoto S Azuma A Kudoh S Fukuchi Y Ouchi Y Matsuse T Association of Gc-globulin variation with susceptibility to COPD and diffuse panbronchiolitis Eur Respir J 2001 18 5 753 7 10.1183/09031936.01.00094401 11757623

[B81] Schellenberg D Paré PD Weir TD Spinelli JJ Walker BA Sandford AJ Vitamin D binding protein variants and the risk of COPD Am J Respir Crit Care Med 1998 157 3 Pt 1 957 61 9517617 10.1164/ajrccm.157.3.9706106

[B82] Wood AM Needham M Simmonds MJ Newby PR Gough SC Stockley RA Phenotypic differences in alpha 1 antitrypsin-deficient sibling pairs may relate to genetic variation COPD 2008 5 6 353 9 10.1080/15412550802522320 19353349

[B83] Yim S Dhawan P Ragunath C Christakos S Diamond G Induction of cathelicidin in normal and CF bronchial epithelial cells by 1,25-dihydroxyvitamin D(3) J Cyst Fibros 2007 6 6 403 10 10.1016/j.jcf.2007.03.003 17467345 PMC2099696

[B84] Trinchieri G Sher A Cooperation of Toll-like receptor signals in innate immune defence Nat Rev Immunol 2007 7 3 179 90 10.1038/nri2038 17318230

[B85] Bhalla AK Amento EP Clemens TL Holick MF Krane SM Specific high-affinity receptors for 1,25-dihydroxyvitamin D3 in human peripheral blood mononuclear cells: presence in monocytes and induction in T lymphocytes following activation J Clin Endocrinol Metab 1983 57 6 1308 10 10.1210/jcem-57-6-1308 6313738

[B86] Chen S Sims GP Chen XX Gu YY Chen S Lipsky PE Modulatory effects of 1,25-dihydroxyvitamin D3 on human B cell differentiation J Immunol 2007 179 3 1634 47 17641030 10.4049/jimmunol.179.3.1634

[B87] Lemire JM Adams JS Kermani-Arab V Bakke AC Sakai R Jordan SC 1,25-Dihydroxyvitamin D3 suppresses human T helper/inducer lymphocyte activity in vitro J Immunol 1985 134 5 3032 5 3156926

[B88] Penna G Adorini L 1 Alpha,25-dihydroxyvitamin D3 inhibits differentiation, maturation, activation, and survival of dendritic cells leading to impaired alloreactive T cell activation J Immunol 2000 164 5 2405 11 10679076 10.4049/jimmunol.164.5.2405

[B89] Barnes PJ Adcock IM Glucocorticoid resistance in inflammatory diseases Lancet 2009 373 9678 1905 17 10.1016/S0140-6736(09)60326-3 19482216

[B90] Sin DD Man JP Man SF The risk of osteoporosis in Caucasian men and women with obstructive airways disease Am J Med 2003 114 1 10 4 10.1016/S0002-9343(02)01297-4 12543283

[B91] Incalzi RA Caradonna P Ranieri P Basso S Fuso L Pagano F Ciappi G Pistelli R Correlates of osteoporosis in chronic obstructive pulmonary disease Respir Med 2000 94 11 1079 84 10.1053/rmed.2000.0916 11127495

[B92] Ferguson GT Calverley PM Anderson JA Jenkins CR Jones PW Willits LR Yates JC Vestbo J Celli B Prevalence and Progression of Osteoporosis in Patients With COPD. Results From TORCH Chest 2009 136 6 1456 65 10.1378/chest.08-3016 19581353

[B93] Graat-Verboom L Wouters EF Smeenk FW van den Borne BE Lunde R Spruit MA Current status of research on osteoporosis in COPD: a systematic review Eur Respir J 2009 34 1 209 18 10.1183/09031936.50130408 19567604

[B94] Franco CB Paz-Filho G Gomes PE Nascimento VB Kulak CA Boguszewski CL Borba VZ Chronic obstructive pulmonary disease is associated with osteoporosis and low levels of vitamin D Osteoporos Int 2009 20 11 1881 7 10.1007/s00198-009-0890-5 19300892

[B95] Riancho JA González Macías J Del Arco C Amado JA Freijanes J Antón MA Vertebral compression fractures and mineral metabolism in chronic obstructive lung disease Thorax 1987 42 12 962 6 10.1136/thx.42.12.962 3438885 PMC461059

[B96] Shane E Silverberg SJ Donovan D Papadopoulos A Staron RB Addesso V Jorgesen B McGregor C Schulman L Osteoporosis in lung transplantation candidates with end-stage pulmonary disease Am J Med 1996 101 3 262 9 10.1016/S0002-9343(96)00155-6 8873487

[B97] de Batlle J Romieu I Antó JM Mendez M Rodríguez E Balcells E Ferrer A Gea J Rodriguez-Roisin R Garcia-Aymerich J The PAC-COPD Study Group. Dietary habits of firstly admitted Spanish COPD patients Respir Med 2009 103 12 1904 10 10.1016/j.rmed.2009.06.001 19564102

[B98] McManus TE Marley AM Baxter N Christie SN Elborn JS O'Neill HJ Coyle PV Kidney JC High levels of Epstein-Barr virus in COPD Eur Respir J 2008 31 6 1221 6 10.1183/09031936.00107507 18287127

[B99] Grant WB A possible role for Epstein-Barr virus infection in COPD? Eur Respir J 2008 32 5 1412 3 10.1183/09031936.00098608 18978144

[B100] Liu PT Stenger S Li H Wenzel L Tan BH Krutzik SR Ochoa MT Schauber J Wu K Meinken C Kamen DL Wagner M Bals R Steinmeyer A Zügel U Gallo RL Eisenberg D Hewison M Hollis BW Adams JS Bloom BR Modlin RL Toll-like receptor triggering of a vitamin D-mediated human antimicrobial response Science 2006 311 5768 1770 3 10.1126/science.1123933 16497887

[B101] Kung T Springer J Doehner W Anker SD von Haehling S Novel treatment approaches to cachexia and sarcopenia: highlights from the 5th Cachexia Conference Expert Opin Investig Drugs 2010 19 4 579 85 10.1517/13543781003724690 20367196

[B102] Deruelle F Baron B Vitamin C: is supplementation necessary for optimal health? J Altern Complement Med 2008 14 10 1291 8 10.1089/acm.2008.0165 19032072

[B103] Jacob RA Assessment of human vitamin C status J Nutr 1990 120 Suppl 11 1480 5 2243292 10.1093/jn/120.suppl_11.1480

[B104] Dutta A Dutta SK Vitamin E and its role in the prevention of atherosclerosis and carcinogenesis: a review J Am Coll Nutr 2003 22 4 258 68 12897039 10.1080/07315724.2003.10719302

[B105] Takkouche B Regueira-Méndez C García-Closas R Figueiras A Gestal-Otero JJ Intake of vitamin C and zinc and risk of common cold: a cohort study Epidemiology 2002 13 1 38 44 10.1097/00001648-200201000-00007 11805584

[B106] Heimer KA Hart AM Martin LG Rubio-Wallace S Examining the evidence for the use of vitamin C in the prophylaxis and treatment of the common cold J Am Acad Nurse Pract 2009 21 5 295 300 10.1111/j.1745-7599.2009.00409.x 19432914 PMC7166744

[B107] Guenegou A Boczkowski J Aubier M Neukirch F Leynaert B Interaction between a heme oxygenase-1 gene promoter polymorphism and serum beta-carotene levels on 8-year lung function decline in a general population: the European Community Respiratory Health Survey (France) Am J Epidemiol 2008 167 2 139 44 10.1093/aje/kwm282 17971338

[B108] Hirayama F Lee AH Binns CW Zhao Y Hiramatsu T Tanikawa Y Nishimura K Taniguchi H Do vegetables and fruits reduce the risk of chronic obstructive pulmonary disease? A case-control study in Japan Prev Med 2009 49 2-3 184 9 10.1016/j.ypmed.2009.06.010 19555711

[B109] Fimognari FL Loffredo L Di Simone S Sampietro F Pastorelli R Monaldo M Violi F D'Angelo A Hyperhomocysteinaemia and poor vitamin B status in chronic obstructive pulmonary disease Nutr Metab Cardiovasc Dis 2009 19 9 654 9 10.1016/j.numecd.2008.12.006 19282159

[B110] Hirayama F Lee AH Binns CW Taniguchi H Dietary supplementation by Japanese patients with chronic obstructive pulmonary disease Complement Ther Med 2009 17 1 37 43 10.1016/j.ctim.2008.02.007 19114227

[B111] Daga MK Chhabra R Sharma B Mishra TK Effects of exogenous vitamin E supplementation on the levels of oxidants and antioxidants in chronic obstructive pulmonary disease J Biosci 2003 28 1 7 11 10.1007/BF02970125 12682418

[B112] Nadeem A Raj HG Chhabra SK Effect of vitamin E supplementation with standard treatment on oxidant-antioxidant status in chronic obstructive pulmonary disease Indian J Med Res 2008 128 6 705 11 19246793

[B113] Wu TC Huang YC Hsu SY Wang YC Yeh SL Vitamin E and vitamin C supplementation in patients with chronic obstructive pulmonary disease Int J Vitam Nutr Res 2007 77 4 272 9 10.1024/0300-9831.77.4.272 18271282

[B114] Paiva SA Godoy I Vannucchi H Fávaro RM Geraldo RR Campana AO Assessment of vitamin A status in chronic obstructive pulmonary disease patients and healthy smokers Am J Clin Nutr 1996 64 6 928 34 8942419 10.1093/ajcn/64.6.928

[B115] Heart Protection Study Collaborative Group. MRC/BHF Heart Protection Study of antioxidant vitamin supplementation in 20,536 high-risk individuals: a randomised placebo-controlled trial Lancet 2002 360 9326 23 33 10.1016/S0140-6736(02)09328-5 12114037

[B116] Pocobelli G Peters U Kristal AR White E Use of supplements of multivitamins, vitamin C, and vitamin E in relation to mortality Am J Epidemiol 2009 170 4 472 83 10.1093/aje/kwp167 19596711 PMC2727181

[B117] Aloia JF Li-Ng M Re: epidemic influenza and vitamin D Epidemiol Infect 2007 135 7 1095 6; author reply 1097-8 10.1017/S0950268807008308 17352842 PMC2870688

[B118] Liu BA McGeer A McArthur MA Simor AE Aghdassi E Davis L Allard JP Effect of multivitamin and mineral supplementation on episodes of infection in nursing home residents: a randomized, placebo-controlled study J Am Geriatr Soc 2007 55 1 35 42 10.1111/j.1532-5415.2006.01033.x 17233683

[B119] Graat JM Schouten EG Kok FJ Effects of daily vitamin E and multivitamin-mineral supplementation on acute respiratory tract infections in elderly persons: a randomized controlled trial JAMA 2002 288 6 715 721 10.1001/jama.288.6.715 12169075

